# Bioelectrical Impedance Analysis and How it Correlates to Intracardiac Hemodynamics in Patients with Congenital Heart Disease

**DOI:** 10.1007/s00246-025-03840-6

**Published:** 2025-04-02

**Authors:** David A. Katz, Zhiqian Gao, Hannah Cope, Sarosh P. Batlivala, Clifford Chin, Alexander R. Opotowsky, Adam W. Powell

**Affiliations:** 1 Department of Pediatrics, University of Cincinnati College of Medicine, Cincinnati, OH USA; 2 Heart Institute, Cincinnati Children’s Hospital Medical Center, 3333 Burnet Avenue, MLC 2003, Cincinnati, OH 45229 USA; 3 Cincinnati Adult Congenital Heart Disease Program, Cincinnati, OH USA; 4 Heart Institute Research Core (HIRC), Cincinnati, OH USA

**Keywords:** Hemodynamics, Bioelectrical impedance analysis, Fluid status, Heart failure, Edema index

## Abstract

Bioelectrical impedance analysis (BIA) is a noninvasive tool that can estimate volume status using fluid compartment ratios. Previous studies have demonstrated that BIA can be used to help manage heart failure using the edema index (EI), which is the ratio of extracellular water (ECW) to total body water (TBW). This study set out to better define the relationship between BIA fluid compartment estimations and invasive hemodynamic measurements, in the context of pediatric and congenital heart disease. 52 individuals underwent 59 elective catheterizations and BIA. Data from the BIAs were compared with the hemodynamic catheterization data. The median age at the time of catheterization was 16.6 [13.5, 19.6] years (63% < 18 years-old), and 29% were female. In multivariable analysis, EI (β = 103.5 ± 47.9, p = 0.04), body mass index (BMI) (β = 0.16 ± 0.07, p = 0.02), and current Fontan circulation (β = 3.06 ± 0.96, p = 0.002) were statistically significant predictors of pulmonary capillary wedge pressure (PCWP). Individuals with an EI ≥ 0.39 had a statistically significant higher PCWP compared to those with an EI < 0.39 (12 [11, 17] vs. 10 [8, 12], = 0.05), with an area under the curve (AUC) of 0.76 (95% CI 0.65, 0.87). There was no statistically significant mean difference between the pre-catheterization and either post-catheterization EI (− 0.0001 mean difference (0% change), p = 0.92), or body fat mass (BFM) (+ 0.035 mean difference (0.2% change), p = 0.81). This study suggests that BIA, and more specifically EI, can noninvasively provide valuable information about hemodynamic data. BIA provides a quick, easy, non-invasive method for assessing and managing the volume status in individuals with congenital heart disease.

## Introduction

Accurate determination of fluid status is critical to appropriate patient management. Noninvasive evaluations to provide guidance for clinical decision-making include physical exam, body mass, intake, and output recordings, as well as reported symptoms. While these each provide important data for clinical decision making, they all have limited accuracy leading to inaccurate fluid status assessment that can cause adverse outcomes [1]. Bioelectrical impedance analysis (BIA) noninvasively estimates body composition and has been used to estimate volume status using fluid compartment ratios. Unlike dual energy x-ray absorptiometry (DEXA) [2], BIA does this without exposure to ionizing radiation. Among BIA markers, edema index (EI) is the ratio of extracellular water (ECW) to total body water (TBW), and has been shown to be a predictor of survival [3, 4].

Elevated, or increasing, EI is associated with poor outcomes for individuals being managed for heart failure [5, 6]. Further, one study found that EI-guided heart failure treatment, using a threshold EI of 0.39, led to improved outcomes and fewer health events [7]. These studies demonstrate that EI can be used to assess and titrate fluid management for acquired heart failure in adults. Little work has been done to understand the utility of BIA in patients with pediatric and congenital heart disease. One study demonstrated that the EI of patients with congenital heart disease was higher in those with worse heart failure symptoms, EI significantly correlated with brain natriuretic peptide (BNP), and an EI > 0.386 at discharge (from a heart failure admission) was associated with increased risk of a future heart failure related admission [8].

Correlations between EI and invasive hemodynamic measurements (the gold standard of fluid status assessment in heart failure) are not well defined. Elucidating the relationship between BIA and invasive hemodynamics may lead to validation of a non-invasive method for early identification of patients who are fluid overloaded, potentially preventing further adverse outcomes related to decompensated heart failure. The objective of this study was to assess the association between BIA and invasive hemodynamic measurements, primarily EI and pulmonary capillary wedge pressure (PCWP). We hypothesized that there is a positive correlation between EI and PCWP, as well as a significant difference in PCWP between individuals with an EI < 0.39 and those with an EI ≥ 0.39.

## Methods

The data analyzed for this study are available to other researchers upon request from the corresponding author. We enrolled 66 individuals between October 2023 and March 2024 who were having elective catheterizations at Cincinnati Children’s Hospital Medical Center (CCHMC). The current study was approved by the CCHMC Institutional Review Board. Written informed consent was obtained from each participant 18 years of age or older, or their parental caregiver if under 18 years of age. Written assent was obtained from participants less than 18 years of age.

Demographic and clinical data were obtained via a detailed review of all available health records. Demographic variables analyzed included: age at the time of catheterization, sex, race, cardiac diagnosis, current Fontan circulation, history of orthotopic heart transplant (OHT), and New York Heart Association Functional Class (NYHA FC).

Individuals recruited were already referred to the catheterization laboratory at CCHMC for a clinically indicated elective catheterization. Exclusion criteria included individuals < 8 years of age, those who were critically ill, referred for an acute procedure, or those with an implantable cardioverter defibrillator (ICD) and/or pacemaker. Individuals were enrolled and consented in the CCHMC Cardiology Anesthesia and Recovery Unit (CARU) prior to elective catheterization that day. Once enrolled, BIA was performed using the InBody570™ (InBody USA, Cerritos, CA, USA) machine in the CARU. A second BIA was planned to be performed in the CARU after the catheterization procedure and just prior to discharge home, when the individual was stable from a cardiovascular and bleeding perspective.

Of the 66 individuals that were enrolled, there were 59 encounters (from 52 individuals) that were included for analysis. Encounters that were included for analysis had at least a pre-catheterization BIA, followed by a hemodynamic catheterization, were included in the overall analysis. The subset of encounters with both pre- and post-catheterization BIA underwent a secondary analysis. Those that did not undergo a post-catheterization BIA were either admitted to the hospital after catheterization or the CARU staff were unable to perform the test due to other clinical responsibilities. The enrolled individuals that were not included for analysis either: did not undergo a pre-catheterization BIA, did not have hemodynamics obtained during the catheterization, or the catheterization was canceled after enrollment. The clinical CARU staff were instructed on proper acquisition of the BIA utilizing the InBody570™ and performed both the pre- and post-catheterization BIAs.

### Statistical Analysis

Continuous variables are presented as median [25th–75th percentile] for non-normally distributed variables and mean ± standard deviation (SD) for normally distributed variables. Linear regression analysis was performed to determine significant correlations between predictor variables and mean PCWP. Multivariable linear regression analysis was used to evaluate associations between variables to predict PCWP. Covariates included in the multivariable model were variables with a P value < 0.1 in univariate analysis, and backward selection based on P value > 0.05 (in the multivariable analysis). A two-tailed P value of ≤ 0.05 was considered statistically significant. A paired t-test was used to analyze the subset of individuals that had pre- and post-catheterization BIAs performed. Statistical analyses were performed using JMP® (version 14, SAS Institute Inc., Cary, NC), and SAS version 9.4 (SAS Institute Inc., Cary, NC).

## Results

### Demographics and Clinical Characteristics

A total of 52 individuals were enrolled in the study, 7 of which underwent more than one hemodynamic catheterization during the study period (5 individuals with 2 encounters, and 2 individuals with 3 encounters), leading to a total of 59 encounters in the data analysis. Table 1 outlines the demographics and clinical characteristics of our cohort. The median age at catheterization was 16.6 [13.5, 19.6] years (63% < 18 years-old), 29% were female, and 24% were African American. Most of the patients (59%, n = 35) were status post orthotopic heart transplant (OHT), while 9 individuals (15%) had current Fontan circulation. Only 8 individuals (14%) had a NYHA FC ≥ 2.

**Table 1:** Demographic and Clinical Characteristics

Total encounters (n)	59
Age at BIA (years)	16.6 [13.5, 19.6]
Age < 18 years-old	63% (n = 37)
Sex (% female)	29% (n = 17)
Race (% African American)	24% (n = 14)
Cardiac diagnosis (%)	
s/p OHT	59% (n = 35)
Other	20% (n = 12)
Current Fontan circulation	15% (n = 9)
TOF	4% (n = 2)
CAA	2% (n = 1)
NYHA FC ≥ 2 (%)	14% (n = 8)

Continuous variables presented as median [25th, 75th percentiles]; categorical variables presented as % (n). 7 individuals had more than one BIA/cath (5 with 2 encounters, 2 with 3 encounters). Other diagnoses include: aortic stenosis, atrial septal defect, Barth syndrome, bicuspid aortic valve, congenital diaphragmatic hernia, cystic fibrosis (lung transplant evaluation), dysplastic pulmonary valve, interrupted aortic arch, pulmonary hypertension, and superior sinus venosus defect.

*BIA* bioelectrical impedance analysis; *CAA* coronary artery anomaly; *OHT* orthotopic heart transplant; *NYHA FC* New York Heart Association Functional Class; *TOF* Tetralogy of Fallot

### BIA Data

Table 2 describes the body composition breakdown for these encounters obtained by BIA, including a median pre-catheterization EI of 0.38 ± 0.007. There was a subset of 20 encounters which included both pre- and post-catheterization BIA assessments. The mean duration between pre- and post-catheterization BIAs was 6.97 ± 2.11 h and the median total IV volume received during the catheterizations was 515 [425, 600] mL. Table 3 compares these pre- and post-catheterization body composition estimations, including body weight (BW), TBW, ECW, intracellular water (ICW), EI, and body fat mass (BFM). There was a significant mean difference for each individual’s pre- and post-catheterization body composition, but there was no significant mean difference between the pre- and post-catheterization EI (-0.0001 mean difference (0% change), p = 0.92), nor BFM (+ 0.035 mean difference (0.2% change), p = 0.81).

**Table 2:** Pre-Catheterization Bioelectrical Impedance Data for All Encounters

n = 59	Pre-Cath BIA
BW (kg)	59.9 ± 22.7
TBW (L)	33.2 ± 11.1
ECW (L)	12.6 ± 4.1
ICW (L)	20.6 ± 7.0
EI (ECW/TBW)	0.38 ± 0.007
BFM (kg)	12.2 [6.4, 21.4]

Normally distributed variables are presented as mean ± standard deviation, whereas non-normally distributed variables are presented as median [25th, 75th percentiles]. There was a median of 3.05 [2.20, 3.92] hours in between the pre-catheterization BIA and the hemodynamic measurements. There were 7 individuals that had more than one BIA/cath (5 individuals with 2 encounters, 2 individuals with 3 encounters)

*Cath* catheterization; *BIA* bioelectrical impedance; *BW* body weight; *TBW* total body water; *ECW* extracellular water; *ICW* intracellular water; *EI* edema index; *BFM* body fat mass

**Table 3:** Pre- and Post-Catheterization Bioelectrical Impedance Data for Subset (n = 20) with Repeated Measurements

n = 20	Pre-Cath	Post-Cath	Mean difference (% change)	*P*
BW (kg)	63.4 ± 26.4	64.1 ± 26.6	+ 0.66 (1.0%)	**0.0002**
TBW (L)	33.5 ± 12.5	33.9 ± 12.6	+ 0.42 (1.2%)	**0.005**
ECW (L)	12.7 ± 4.7	12.8 ± 4.7	+ 0.16 (1.2%)	**0.009**
ICW (L)	20.8 ± 7.8	21.1 ± 7.9	+ 0.27 (1.3%)	**0.007**
EI (ECW/TBW)	0.38 ± 0.006	0.38 ± 0.006	-0.0001 (0%)	0.92
BFM (kg)	17.7 ± 10.9	17.7 ± 11.0	+ 0.035 (0.2%)	0.81

variables are presented as mean ± standard deviation. Mean difference represents Post-Cath BIA subtracted by Pre-Cath BIA for each variable. P represents the p-values of the paired t-Test between Pre-Cath BIA and Post-Cath BIA for each variable. *P* < 0.05 are presented in bold font. The mean time between BIAs was 6.97 ± 2.11 h, and the median total IV volume received during the catheterizations was 515 [425, 600] mL. There was one participant who underwent two catheterizations, which are each considered independently

*Cath* catheterization; *BIA* bioelectrical impedance; *BW* body weight; *TBW* total body water; *ECW* extracellular water; *ICW* intracellular water; *EI* edema index; *BFM* body fat mass; *IV* intravenous

### Hemodynamic Data and Correlations

Individuals were nil per os (NPO) for a mean of 9.2 ± 3.2 h prior to catheterization. The median time from pre-catheterization BIA to hemodynamic measurements was 3.05 [2.20, 3.92] hours. The median PCWP for the cohort was 10 [8.5, 12] mmHg and the ratio of pulmonary blood flow to systemic blood flow (Q_P_:Q_S_) was 1:1. Complete hemodynamic data for the cohort is outlined in Table 4. Univariate analysis can be seen in Table 5, with mean PCWP as the dependent continuous variable. Age at pre-catheterization BIA (p = 0.05, *r*^*2*^ = 0.07), body mass index (BMI) (p = 0.01, *r*^*2*^ = 0.12), and percent body fat (p = 0.004, *r*^*2*^ = 0.14) were the variables associated with PCWP (p < 0.05).

**Table 4:** Hemodynamic Data

Total catheterizations	n = 59
NPO time (hours)	9.2 ± 3.2
Mean PA pressure (mmHg)	15 [14, 18]
Mean PCWP (mmHg)	10 [8.5, 12]
TPG (mmHg)	5 [3.5, 7]
Q_Pi_ (L/min/m^2^)	3.7 [3.2, 4.4]
Q_Si_ (L/min/m^2^)	3.7 [3.2, 4.3]
Q_P_:Q_S_	1 [1]
PVR* (WU)*	0.8 [0.6, 1.3]

Normally distributed variables are presented as mean ± standard deviation, whereas non-normally distributed variables are presented as median [25th, 75th percentiles]. There were 7 individuals that had more than one BIA/cath (5 individuals with 2 encounters, 2 individuals with 3 encounters)

*NPO* nil per os; *PA* pulmonary artery; *PCWP* pulmonary capillary wedge pressure; *TPG* transpulmonary gradient; *Q*_*Pi*_ pulmonary blood flow indexed to body surface area; *Q*_*si*_ systemic cardiac output indexed to body surface area; *PVR* pulmonary vascular resistance; *WU* Wood Units

**Table 5:** Correlation analysis of Pulmonary Capillary Wedge Pressure

Dependent variable	Predictor variables	*P*	*r*^*2*^
Mean PCWP (mmHg)	Age	**0.05**	0.07
	Body Weight (kg)	0.10	0.05
	BMI (kg/m2)	**0.01**	0.12
	BSA (m2)	0.21	0.03
	TBW (L)	0.48	0.01
	ECW (L)	0.41	0.01
	ICW (L)	0.52	0.01
	Percent BF (%)	**0.004**	0.14
	EI	0.08	0.05

This table represents univariate analyses between the dependent (mean PCWP) and predictor continuous variables. Linear regression analysis was performed and *r*^*2*^ represents the coefficient of determination. *P* represents p-values, which were considered statistically significant (and highlighted in bold) if ≤ 0.05

*PCWP* pulmonary capillary wedge pressure; *BMI* body mass index; *BSA* body surface area; *TBW* total body water; *ECW* extracellular water; *ICW* intracellular water; *BF* body fat; *EI* edema index

In multivariable analysis, three variables were identified as independently predictive of PCWP: 1) EI (β = 103.5 ± 47.9, p = 0.04), 2) BMI (β = 0.16 ± 0.07, p = 0.02), and 3) current Fontan circulation (β = 3.06 ± 0.96, p = 0.002). This multivariable model explained 33% of the variability in PCWP (r^2^ = 0.33, r = 0.57), consistent with a moderately strong relationship (Table 6). Variables significant (p < 0.1) in the univariate analysis (BMI, EI, current Fontan circulation, age, BSA, and PBF) were selected for multivariate analysis for correlation with mean PCWP; age, BSA, and PBF were removed during a multivariable model backward selection process (p > 0.05 in multivariable analysis).

**Table 6:** Multivariable analyses of Pulmonary Capillary Wedge Pressure

Dependent variable	Predictor variables	β ± SE	*P*	*Model r*^*2*^
	BMI	0.16 ± 0.07	**0.02**	
	EI (ECW/TBW)	103.5 ± 47.9	**0.04**	
Mean PCWP	Fontan (Y or N)	3.06 ± 0.96	**0.002**	0.33

This table demonstrates that with PCWP as the dependent variable, BMI, pre-cath EI, and Fontan (yes or no) each independently provide statistically significant information to predict PCWP. The covariates included were chosen due to their p-value being < 0.1 in univariate analysis. P represents p-values for each β coefficient. *P* ≤ 0.05 are presented in bold font. *r*^*2*^ is for the entire multivariable model

*EI* edema index; *ECW* extracellular water; *TBW* total body water; *BMI* body mass index; *PCWP* pulmonary capillary wedge pressure; *Y* yes; *N* No; *BIA* bioimpedance analysis

**Fig. 1:**
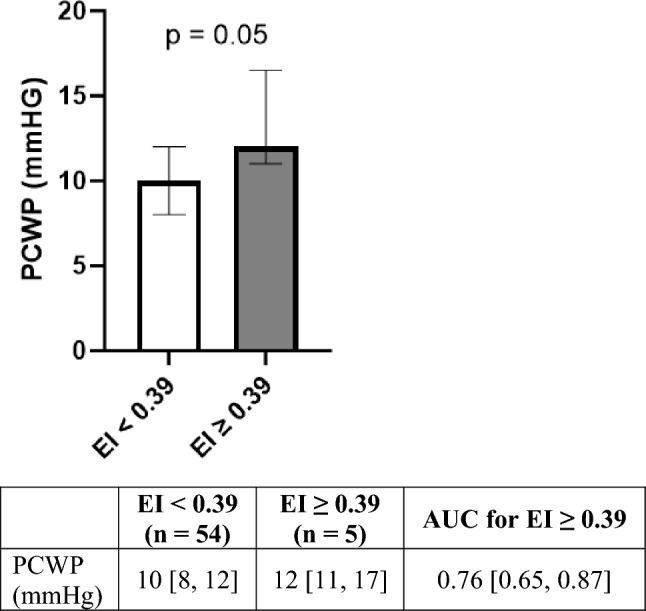
Pulmonary Capillary Wedge Pressure (PCWP) by Edema Index (EI), Normal (< 0.39) vs. Elevated (≥ 0.39). This figure compares the PCWP of those with an EI < 0.39 (n = 54) with those with EI ≥ 0.39 (n = 5). Error bars represent the interquartile range for each group (25th and 75th percentiles). Wilcoxon rank sums test was used to compare the groups and p = 0.05. AUC for EI ≥ 0.39 was calculated using ROC and is presented with Wald 95% confidence intervals. There were 7 individuals that had more than one BIA/cath (5 individuals with 2 encounters, 2 individuals with 3 encounters). *EI* edema index; *PCWP* pulmonary capillary wedge pressure; *AUC* area under the curve; *ROC* receiver operating characteristic; *BIA* bioelectrical impedance

Individuals with an EI ≥ 0.39, had a significantly higher PCWP compared to those with an EI < 0.39 (12 [11, 17] vs. 10 [8, 12], = 0.05, Fig. 1), with an area under the curve (AUC) of 0.76 (95% CI 0.65, 0.87).

## Discussion

This prospective study examined the association between BIA body composition assessments and intracardiac hemodynamic pressure measurements. The results suggest that (1) EI is positively associated with PCWP, accounting for current Fontan circulation and BMI; (2) individuals with EI ≥ 0.39 on average have statistically significant higher PCWP compared to those with an EI < 0.39; (3) the EI cutoff applied to other populations, 0.39, appears reasonably well calibrated to identify an elevated PCWP in pediatric and congenital heart disease; and (4) BIA appears able to identify small changes in fluid status as evidenced by change in pre- and post-catheterization BIA fluid estimates.

BIA has been established as a non-invasive tool to help determine an individual's fluid status in the ambulatory and inpatient settings [9]. This has been applied to patients with heart failure, but there has not been a direct comparison between BIA and intracardiac hemodynamics. In our study, BIA was used to estimate body composition and fluid status in the pre-procedure holding area (CARU) just before intracardiac pressure measurements were obtained via catheterization. While EI had a trend toward a statistically significant correlation with mean PCWP in univariate analysis (p = 0.08, r^2^ = 0.05), multivariable analysis demonstrated that EI, along with BMI and current Fontan circulation, was a statistically significant predictor of mean PCWP (β = 103.5 ± 47.9, p = 0.04). When assessing the relationship between EI and PCWP, accounting for both BMI and current Fontan circulation appears important. To maximize recruitment, we allowed for a wide age range and diagnosis. As such, our cohort included both pediatric and adult individuals, with a wide age range (8–41 years of age). Given that children on average have lower BMI, there was also a wide range in BMI (13–37 kg/m^2^), which affects PCWP [10]. The diagnoses ranged from post-transplant to biventricular heart disease to single ventricle Fontan.

In terms of the Fontan circulation, this subgroup is prone to early diastolic dysfunction secondary to many factors, including increased ventricular mass-volume ratio, hypoxemia, and myocardial fibrosis [11–14]. It is notable that for the same EI, those with a Fontan circulation, on average, had a PCWP 3 mmHg higher than for those without a Fontan. Put another way, for a given elevation in PCWP, those with a Fontan have less severe fluid redistribution from the intracellular to extracellular fluid compartments. One might expect the converse, since systemic venous pressure would be higher relative to any given PCWP, but those with a Fontan circulation are in a state of chronic compensation involving marked reduction in venous compliance and elevated microvascular filtration pressures and thresholds for edema [15].

Our study demonstrated that if an individual had an EI greater than or equal to 0.39, their PCWP was statistically significantly higher than if their EI was less than 0.39. Also, our study showed that using an EI cutoff of 0.39 could reasonably predict PCWP. Invasive hemodynamics are the gold standard for determining intravascular volume, and PCWP typically becomes elevated prior to symptom onset [16, 17]. Symptomatic edema secondary to heart failure is an end-product of low cardiac output, leading to decreased renal perfusion, and increased renal sodium and water reabsorption [18]. Peripheral edema, however, may not be palpable until individuals increase their ECW by 2.5-5L [19, 20]. Using BIA, specifically EI, our study showed that an increase in ECW (and thus EI) can be detected prior to the onset of symptomatic edema. All subject participants were clinically well when presenting for their elective catheterization, and none of them were in decompensated heart failure. This correlates with the fact that elevated or increasing EI during hospitalizations leads to increased morbidity and mortality for patients with heart failure [5, 21].

Supporting the reliability of the InBody machine used in this study, we performed BIA on a subset of 20 individuals (1 participant underwent 2 catheterizations; each were considered independently) before and after catheterization. There was a statistically significant increase in all of the body compartment estimations one would expect to increase after receiving an intravenous (IV) volume load during the catheterization, and then eating and drinking after the catheterization (the post-catheterization BIA was obtained directly prior to discharge, such that the patient had met discharge criteria which include eating, drinking, walking, and voiding). The body compartment estimates that increased included: BW, TBW, ECW, and ICW, with a mean increase ranging from 1–1.3%. Conversely, BFM did not change from pre- to post-catheterization, which makes sense since BFM should not change over this time course (mean of 7 h between BIAs), regardless of fluid status.

## Limitations

These findings must be interpreted in the context of a relatively small sample size, which limits statistical power. While the intention was to enroll between 80 and 100 individuals, this was not met due to time and logistical constraints. Post-catheterization BIAs were intended to be performed for every individual, but this was not accomplished due to a variety of factors including admission to the inpatient unit, bleeding concerns, and availability of the CARU staff due to clinical responsibilities. All the individuals in this study were referred for elective catheterization; therefore, the cohort was relatively healthy, with only a small subset having clinically elevated EI or PCWP, limiting statistical power.

## Conclusions

These data suggest that BIA provides independent noninvasive information about hemodynamic data that otherwise requires invasive catheterization. After adjusting for important clinical associations with BMI and presence of a Fontan circulation, EI correlates significantly with PCWP, and EI ≥ 0.39 is suggestive of clinically elevated PCWP. Further research is needed prior to widespread clinical implementation with additional assessment in specific patient subsets, but BIA is a promising adjunct for assessing and managing the volume status in individuals with heart failure. This is especially notable since BIA is not only noninvasive, but also fast and easy to obtain (the InBody570™ (InBody USA, Cerritos, CA, USA) machine takes 50 s to perform a BIA).

## Data Availability

The data collected and analyzed for this study are not openly available to the public. This is meant to protect the sensitive health information of the subjects that were recruited and consented to be in this study. The data is contained within a protected storage drive at Cincinnati Children's Hospital Medical Center, and are possibly available from the corresponding author upon request.

## Abbreviations

AUC: Area under the curve
BIA: Bioelectrical impedance analysis
BFM: Body fat mass
BMI: Body mass index
BW: Body weight
BNP: Brain natriuretic peptide
CARU: Cardiology Admission and Recovery Unit
DCM: Dilated cardiomyopathy
ECW: Extracellular water
ICW: Intracellular water
NYHA FC: New York Heart Association Functional Class
OHT: Orthotopic heart transplant
PCWP: Pulmonary capillary wedge pressure
RCM: Restrictive cardiomyopathy
TBW: Total body water
